# Hepatic insulin sensitivity is improved in high‐fat diet‐fed *Park2* knockout mice in association with increased hepatic AMPK activation and reduced steatosis

**DOI:** 10.14814/phy2.14281

**Published:** 2019-11-13

**Authors:** Lia R. Edmunds, Brydie R. Huckestein, Mario Kahn, Dongyan Zhang, Yanxia Chu, Yingze Zhang, Stacy G. Wendell, Gerald I. Shulman, Michael J. Jurczak

**Affiliations:** ^1^ Division of Endocrinology and Metabolism University of Pittsburgh School of Medicine Pittsburgh Pennsylvania; ^2^ Department of Internal Medicine Yale University School of Medicine New Haven Connecticut; ^3^ Division of Pulmonary, Allergy and Critical Care Medicine Department of Medicine University of Pittsburgh School of Medicine Pittsburgh Pennsylvania; ^4^ Department of Pharmacology and Chemical Biology University of Pittsburgh School of Medicine Pittsburgh Pennsylvania; ^5^ Center for Metabolism and Mitochondrial Medicine University of Pittsburgh School of Medicine Pittsburgh Pennsylvania; ^6^ Department of Cellular and Molecular Physiology Yale University School of Medicine New Haven Connecticut

**Keywords:** AMPK, diacylglycerol, IL6, insulin resistance, liver, mitophagy, Park2, Parkin, PKCε

## Abstract

*Park2* is an E3 ubiquitin ligase known for its role in mitochondrial quality control via the mitophagy pathway. *Park2* KO mice are protected from diet‐induced obesity and hepatic insulin sensitivity is improved in high‐fat diet (HFD)‐fed *Park2* KO mice even under body weight‐matched conditions. In order to better understand the cellular mechanism by which *Park2* KO mice are protected from diet‐induced hepatic insulin resistance, we determined changes in multiple pathways commonly associated with the pathogenesis of insulin resistance, namely levels of bioactive lipid species, activation of the endoplasmic reticulum (ER) stress response and changes in cytokine levels and signaling. We report for the first time that whole‐body insulin sensitivity is unchanged in regular chow (RC)‐fed *Park2* KO mice, and that liver diacylglycerol levels are reduced and very‐long‐chain ceramides are increased in *Park2* KO mice fed HFD for 1 week. Hepatic transcriptional markers of the ER stress response were reduced and plasma tumor necrosis factor‐α (TNFα), interleukin‐6 and −10 (IL6, IL10) were significantly increased in HFD‐fed *Park2* KO mice; however, there were no detectable differences in hepatic inflammatory signaling pathways between groups. Interestingly, hepatic adenylate charge was reduced in HFD‐fed *Park2* KO liver and was associated increased activation of AMPK. These data suggest that negative energy balance that contributed to protection from obesity during chronic HFD manifested at the level of the hepatocyte during short‐term HFD feeding and contributed to the improved hepatic insulin sensitivity.

## Graphical abstract

*Park2* knockout mice‐fed HFD for 1‐week were previously demonstrated to have improved hepatic insulin sensitivity. Here we demonstrate that this phenotype is associated with reduced hepatic triglyceride and diacylglycerol levels, increased very‐long chain ceramides and reductions in markers of endoplasmic reticulum stress. Hepatic AMPK activation was also increased and suggests that the underlying mechanism for improved hepatic insulin sensitivity is multi‐factorial and due to negative energy balance in *Park2* knockout mice.

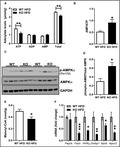

## 1 INTRODUCTION

*PARK2*, or Parkin, was first identified as the mutated gene responsible for autosomal recessive juvenile Parkinsonism in a small cohort of Japanese patients (Kitada et al., 1998). Parkin is now recognized as the most frequently mutated gene in familial Parkinsonism with recessive inheritance, accounting for approximately half of all cases (Bonifati, 2012). Parkin is an E3 ubiquitin ligase and member of the RING‐in‐between‐RING or RING‐HECT hybrid class of E3s (Dove & Klevit 2017) and thus catalyzes the addition of ubiquitin to specific substrate proteins. Parkin is best known for its role in mitochondrial quality control via the mitophagy pathway where it contributes to the degradation of damaged mitochondria (Youle & Narendra, 2011), but Parkin also appears to regulate a number of cellular processes unrelated to mitophagy, such as cell cycle progression, DNA binding, and transcriptional repression (da Costa et al., 2009; Gong et al., 2014). Parkin is expressed in a wide variety of cell types, including adipocytes (Lu et al., 2018), cardiomyocytes (Piquereau et al., 2013), and hepatocytes (Kim et al., 2011), suggesting functional significance outside the central nervous system. In support of this notion, a common feature of most in vivo models of Parkin deficiency is their increased susceptibility to stress in peripheral cell types; myocyte mitochondrial morphology is impaired and wing muscles are atrophied in Parkin homolog null *Drosophilia* (Greene et al., 2003); lipopolysaccharide‐treated *Park2* knockout (KO) mice fail to recover cardiomyocyte mitochondrial respiratory capacity and cardiac contractility (Piquereau et al., 2013); and both acute and chronic exposure to alcohol induces more severe hepatocyte lipid accumulation and inflammation in *Park2* KO mice (Williams, Ni, Ding, & Ding, 2015).

One of the more striking phenotypes described in the *Park2* KO mouse model was their protection from diet‐induced obesity and hepatosteatosis; after six and a half weeks of high‐fat diet (HFD) feeding, *Park2* KO mice weighed 30% less than controls, which was largely attributed to differences in fat mass, and liver fat was also 50% less (Kim et al., 2011). Not surprisingly, HFD‐fed *Park2* KO mice displayed improved glucose and insulin tolerance when compared with obese HFD‐fed wild‐type (WT) mice, but it was unclear whether changes in liver fat and glucose homeostasis after HFD feeding were due to loss of *Park2* or secondary to the protection from obesity (Kim et al., 2011). To address this question, we fed *Park2* KO mice a short‐term, one‐week HFD in order to induce hepatic insulin resistance without major changes in body weight (Costa et al., 2016). Under these conditions, body fat was modestly reduced by 1.2 g or 5% in *Park2* KO mice, but there was no difference in body weight. Hepatic insulin sensitivity, as assessed by hyperinsulinemic euglycemic clamp, was markedly improved in *Park2* KO mice; whereas hyperinsulinemia produced only a 40% reduction in hepatic glucose production in HFD‐fed WT mice, hepatic glucose production was almost completely suppressed (~97%) by insulin in HFD‐fed *Park2* KO mice (Costa et al., 2016). These data demonstrated that *Park2* KO mice were protected against diet‐induced hepatic insulin resistance independent of changes in body weight, but the underlying mechanism was not addressed.

We undertook the studies described here to determine the underlying mechanism for the improved hepatic insulin sensitivity in the HFD‐fed *Park2* KO mice, as well as to address outstanding questions regarding insulin sensitivity in chow‐fed animals. We evaluated key pathways commonly implicated in the pathogenesis of hepatic insulin resistance, including changes in hepatic lipid metabolites, activation of endoplasmic reticulum (ER) stress response, and alterations in inflammatory cytokine levels and signaling pathways downstream of these mechanisms. Overall, we found that hepatic triglyceride and diacylglycerol levels were reduced in *Park2* KO compared with WT mice after short‐term HFD feeding, as well as markers of ER stress. Also, plasma tumor necrosis factor‐α (TNFα), interleukin‐6 (IL6) and interleukin‐10 (IL10) levels were increased in *Park2* KO mice. However, the stress‐activated kinases associated with these pathways were differentially affected in that diacylglycerol‐activated protein kinase C‐ε (PKCε) was reduced in *Park2* KO mouse liver, while ER stress‐associated and inflammatory‐mediated JNK and IKKβ activation were unchanged. Finally, the reduced lipid levels in *Park2* KO mouse livers were associated with increased activation of the cellular energy sensor AMP kinase (AMPK), suggesting that the negative energy balance that contributed to protection from obesity during chronic HFD feeding in the *Park2* KO mice manifested at the level of the hepatocyte during short‐term HFD feeding and contributed to the improved hepatic insulin sensitivity.

## 2 MATERIALS AND METHODS

### 2.1 Animal use and care

**Figure 1:**
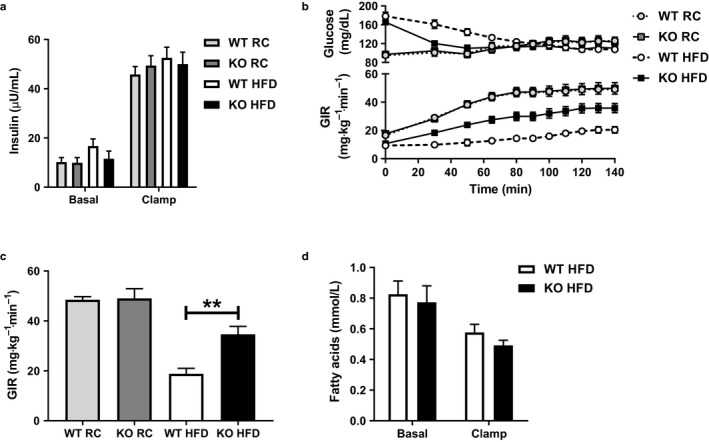
Insulin sensitivity is unchanged in chow‐fed *Park2* KO mice, but improved following short‐term HFD feeding. (a) Basal (fasted) and clamped plasma insulin levels in regular chow (RC)‐ and HFD‐fed mice. (b) Plasma glucose levels and the glucose infusion rate (GIR) required to maintain euglycemia during the clamp. (c) Steady‐state GIR during the last 40 min of the clamp. (d) Basal and clamped plasma fatty acid levels in HFD‐fed mice. Data are the mean ± *SEM* and were analyzed by Student's *t*‐test. **p < .01; *n* = 6–8 mice/group. Data for HFD‐fed mice in panel a, b and c were previously reported (Costa et al., 2016) and are shown here for comparison to RC‐fed mice

**Figure 2:**
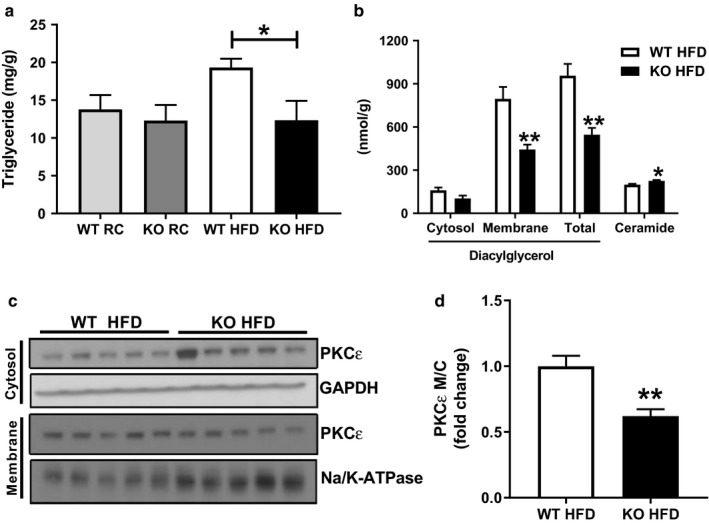
Improved hepatic insulin sensitivity in HFD‐fed *Park2* KO is associated with reduced hepatosteatosis, diacylglycerol levels, and PKCε activation. (a) Liver triglyceride levels in RC‐ and HFD‐fed mice. (b) Liver diacylglycerol (cytosol, membrane‐associated and total) and ceramide levels in HFD‐fed mice. (c) Western blots for PKCε levels in cytosol and membrane fractions from liver of HFD‐fed mice, as well as GAPDH and Na/K‐ATPase loading controls. (d) PKCε activation in liver from HFD‐fed mice expressed as the quantification of the membrane to cytosol ratio of PKCε protein levels shown in C. Data are the mean ± *SEM* and were analyzed by Student's *t*‐test. *p < .05, **p < .01 ***p < .001; *n* = 5–6 mice/group

**Figure 3:**
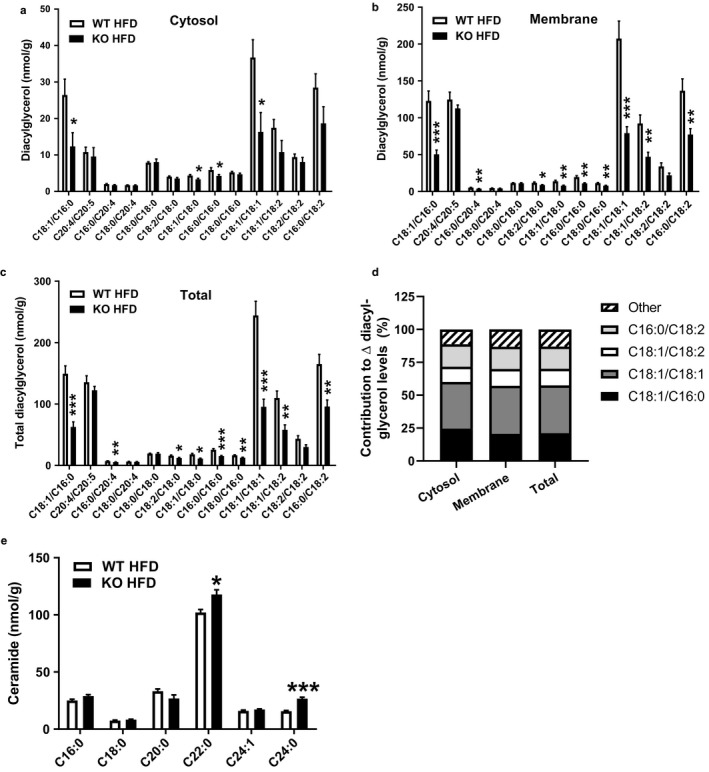
Diacylglycerol acyl chain composition in liver fractions from HFD‐fed *Park2* KO and WT mice. (a) Cytosolic, (b) membrane, and (c) total diacylglycerol acyl chain species in liver of HFD‐fed mice. (d) Percent contribution of individual diacylglycerol species to changes in total levels in cytosol, membrane and total fractions. (e) Ceramide acyl chain composition. Data are the mean ± *SEM* and were analyzed by Student's *t*‐test. *p < .05, **p < .01, ***p < .001, *n* = 5–6 mice/group

**Figure 4:**
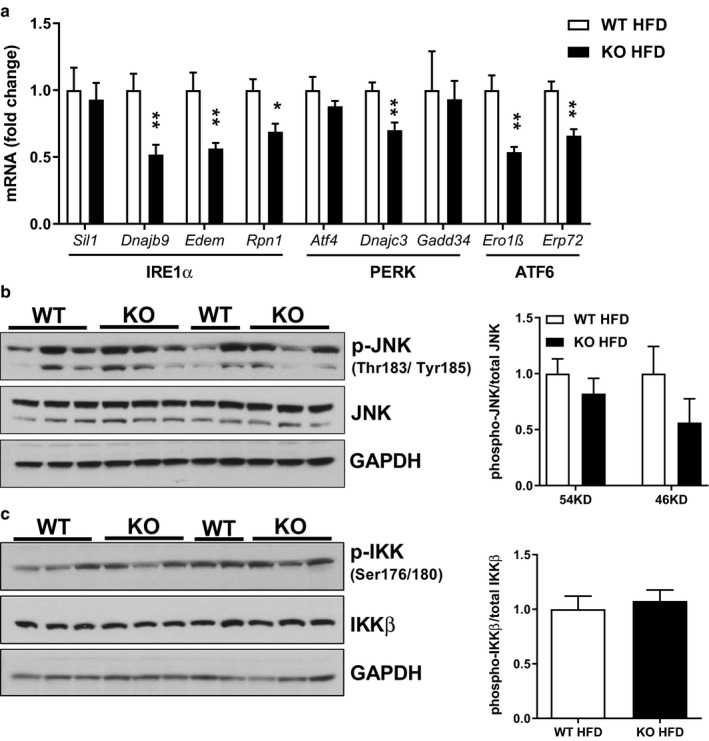
Improved hepatic insulin sensitivity in HFD‐fed *Park2* KO is associated with reduced expression of transcriptional markers of ER stress, but not changes in JNK or IKKβ activation. (a) Liver mRNA levels of markers of ER stress downstream of the three branches of the UPR (IRE1α, PERK and ATF6). (b) Liver protein expression and quantification of blots for phospho‐Thr183/Tyr185 JNK relative to total JNK. (c) Liver protein expression and quantification of blots for phospho‐Ser176/180 IKKβ to total IKKβ. Data are the mean ± *SEM* and were analyzed by Student's *t*‐test. *p < .05, **p < .01; *n* = 5–6 mice/group

**Figure 5:**
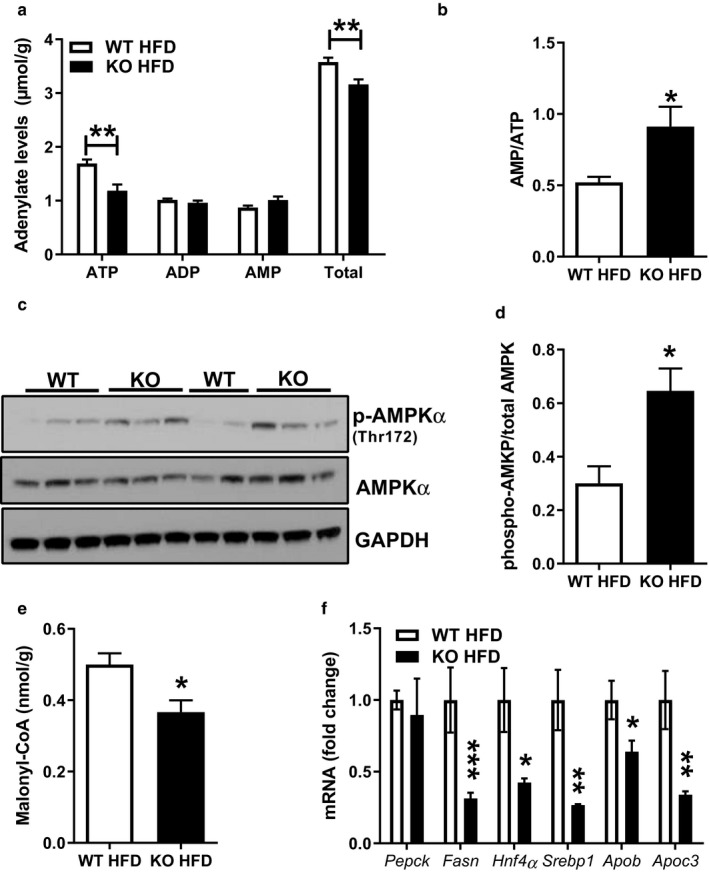
Liver AMPK activation is increased in HFD‐fed *Park2* KO mice. (a) Liver adenylate levels from HFD‐fed mice. (b) Liver AMP to ATP ratio calculated using data in (a). (c) Western blots from liver of HFD‐fed mice for phospho‐AMPKα (Thr172), total AMPKα and GAPDH loading control. (d) Quantification of data in (c). (e) Liver malonyl‐CoA levels. (f) Liver mRNA levels expressed as fold change relative to WT relative expression levels using *Actb* for normalization. Data are the mean ± *SEM* and were analyzed by Student's *t*‐test. *p < .05, **p < .01 ***p < .001; *n* = 5–6 mice/group

Mice were housed and studied at Yale University School of Medicine and the University of Pittsburgh according to guidelines established by the Institutional Animal Care and Use Committees at each institution. Mice were housed at 22 ± 2°C on a 12 hr light, 12 hr dark cycle with free access to food and water. Diets provided were Harlan Teklad 2018S (control rodent chow) and Research Diets D12492 (HFD, 60% kcal fat). WT and *Park2* KO mice on the C57BL6/J background were sourced from the Jackson Laboratory, as previously described (Costa et al., 2016). Mice were between 12 and 14 weeks old at the time of study. Two cohorts of mice were used for the studies; cohort one underwent hyperinsulinemic euglycemic clamps after a 6‐hr morning fast for data shown in Figure 1 (more detail below); and cohort two was euthanized after a 6‐hr morning fast for dissection and collection of plasma and tissue for data reported in Figures 2, 3, 4, 5 and Table 1.

**Table 1:** Plasma cytokine and obesity/diabetes‐related hormone levels in RC‐ and HFD‐fed *Park2* KO and WT mice

Analyte (pg/ml)	WT RC (Average ± *SEM*)	KO RC (Average ± *SEM*)	*p*‐value WT:KO LFD	WT HFD (Average ± *SEM*)	KO HFD (Average ± *SEM*)	*p*‐value WT:KO HFD
IFNγ	0.370 ± 0.117	0.262 ± 0.059	*N*.S.	0.276 ± 0.068	0.178 ± 0.025	*N*.S.
IL2	0.462 ± 0.094	0.385 ± 0.054	*N*.S.	0.514 ± 0.119	0.403 ± 0.046	*N*.S.
IL5	0.944 ± 0.184	1.190 ± 0.282	*N*.S.	1.826 ± 0.807	0.805 ± 0.055	*N*.S.
TNFα	5.48 ± 1.03	5.29 ± 0.32	*N*.S.	4.97 ± 0.43	8.60 ± 0.68	***p* < .01**
IL10	15.13 ± 6.77	9.62 ± 0.99	*N*.S.	7.51 ± 0.54	14.75 ± 0.86	***p* < .001**
IL6	2.58 ± 0.37	5.87 ± 1.19	***p* < .05**	3.80 ± 0.89	40.32 ± 13.18	***p* < .05**
CXCL1	51.96 ± 6.95	56.83 ± 6.45	*N*.S.	59.42 ± 8.91	62.08 ± 8.42	*N*.S.
GLP‐1	2.68 ± 1.91	2.06 ± 0.50	*N*.S.	20.70 ± 17.60	3.30 ± 1.32	*N*.S.
Glucagon	16.26 ± 9.81	12.90 ± 5.05	*N*.S.	39.60 ± 25.92	13.98 ± 4.03	*N*.S.
GIP	105.6 ± 6.0	115.9 ± 10.2	*N*.S.	224.1 ± 56.7	147.4 ± 10.9	*N*.S.
PAI‐1	826.7 ± 61.8	994.5 ± 129.7	*N*.S.	1,088.0 ± 151.8	1,349.3 ± 164.0	*N*.S.
Leptin	1503.6 ± 217.9	526.2 ± 253.5	***p* < .05**	9,527.6 ± 2044.1	533.6 ± 159.4	***p* < .001**
Ghrelin	8,649 ± 1777	14,358 ± 2091	*p* = .071	9,515 ± 4,438	12,500 ± 1988	*N*.S.
Resistin	151,912 ± 28,820	141,808 ± 9,259	*N*.S.	231,133 ± 33,850	161,081 ± 16,254	*p* = .080

Note

Upper portion: Plasma pro‐ and anti‐inflammatory cytokine levels in RC‐ and HFD‐fed mice. Lower portion: Plasma obesity/diabetes‐related hormone levels in RC‐ and HFD‐fed mice. *n* = 5–6 mice/group. Data are the mean ± *SEM* and were analyzed by Student's *t*‐test for RC and HFD groupings and *p*‐values are as shown within the table.

Bold indicates statistical significant values.

### 2.2 Biochemical analyses

Plasma glucose was measured by the glucose oxidase method using the Analox GM9 Glucose Analyzer. Plasma insulin was determined by radioimmunoassay kit (Linco Research) and plasma fatty acids were measured using a spectrophotometric NEFA detection reagent (Wako Diagnostics). Liver triglycerides were determined using the colormetric Infinity Triglyceride Reagent (Fisher Scientific) following tissue homogenization and lipid extraction in chloroform:methanol (2:1), followed by acidification with H_2_SO_4_ and phase separation by centrifugation. Ceramide and diacylglycerol extraction and analysis were performed as described previously (Cantley et al., 2013). Membrane and cytosolic PKCε were measured as previously described (Petersen et al., 2016). Plasma cytokines were measured by electrochemiluminescence using a Diagnostics V‐Plex Proinflammatory Panel I Mouse Kit (Meso Scale Discovery). The plasma obesity/diabetes panel was measured using a Bio‐Plex Pro Mouse Diabetes 8‐Plex Assay (BioRad). All spectrophotometric measurements were performed on a Thermo Scientific Multiskan GO. Liver malonyl‐CoA levels were measured by liquid chromatography mass spectrometry (LC/MS) using an Ultimate 3000 RSLC system connected to a Thermo Fisher Scientific Q Exactive MS (Thermo Fisher Scientific). Briefly, samples were extracted in 80% methanol and purified by silica gel cartridge (Supelco) followed by separation by ion‐pairing reversed phase chromatography by LC‐MS. The MS was equipped with an ESI source and was operated in negative mode using a scan range of 750 *m/z* to 1,250 *m/z*. Analyte identification was confirmed by high‐resolution accurate mass. Liver adenylate levels were measured by capillary electrophoresis (CE)‐MS by Human Metabolome Technologies.

### 2.3 Protein isolation and western blotting

Flash‐frozen liver was pulverized and homogenized in radioimmunoprecipitation assay buffer (150 mM NaCl, 1% (v/v) Triton X‐100, 0.5% (w/v) sodium deoxycholate, 0.1% (w/v) SDS and 50 mM Tris‐HCl, pH 8.0) supplemented with HALT protease inhibitor cocktail (Thermo Fisher). Western blotting electrophoresis was carried out using the ThermoFisher Bolt System, and protein was transferred to a nitrocellulose membrane using BioRad Trans‐blot SD Semi‐dry Transfer Cell. Membranes were probed with the following antibodies and developed on a Licor Odyssey CLx with Licor IRDye goat secondary antibodies. Blots were quantified using Licor Image Studio Software (Ver. 5.2). Antibodies: α1 Na‐K‐ATPase (Abcam ab7671), PKCε (BD Laboratories 610085), GAPDH (Santa Cruz sc‐25778) and from Cell Signaling IKKα/β (pSer176/180; 2697), IKKβ (2370), AMPKα (5831), AMPKα (pThr172; 2535), SAPK/JNK (9252), SAPK/JNK (pThr183/pTyr185; 4668).

### 2.4 RNA isolation, cDNA synthesis and quantitative PCR

RNA was isolated from liver using a QiaShredder plus RNeasy Kit (Qiagen) and then used for cDNA synthesis using a QuantiTect Reverse Transcriptase Kit (Qiagen), both according to manufacturer's instructions. Quantitative PCR was performed using the Applied Biosystems QuantStudio3 RT‐PCR System and PowerUp SYBR Green Master Mix (Thermo Fisher). Primer sequences were designed with IDT Real Time PCR tool and were as follows: *Ero1β* (F CG TCCTTTAAATCCTTTGGCG; R ACACAAACCTTCTAGCCACG) *Erp72* (F ATCATTGGGCTCTTTCAGGG; R AGGAACTTGGCTATTTCAGGG) *ATF4* (F ATGGCGTATTAGAGGCAGC; R CTTTGTCCGTTACAGCAAC AC) *Gadd34* (F GATCGCTTTTGGCAACCAG; R CAGGAGATAGAAGTTGTGGGC) *Dnajc3* (F TGGAGTAAAT GCGGATGTGG; R ACGGTCGCTCTCCTATAGTATG) *Dnajb9* (F AAATAAAAGCCCTGATGCTGAAG; R CCT CTTTGTCCTTTGCCATTG) *Edem* (F CAATGAAGGAGAAGGAGACCC; R GCATCTTCCACATCCCCTATC) *Rpn1* (F TGGATGACTCTGTGGAAATGG; R TCTACAAACCGCATCTTCAGTG) *Sil1* (F GATACAAAGGAGAC CAGGGAAG; R AGTCTGTAGGTTCATTCGCAC) *Fasn* (F CAAGCAGAATTTGTCCACCTTTAA; R TCTCTAG AGGGCTTGCACCAA) *Pepck1* (F GTCACCATCACTTCCTGGAAGA; R GGTGCAGAATCGCGAGTTG) *Hnf4α* (F TGTTTGGTGTGAAGGTCATGATTA; R CAGACGTCCTCCTTTTCTTGTGATA) *Srebp1* (F CCAGAGGGTG AGCCTGACAA; R AGCCTCTGCAATTTCCAGATCT) *Apob* (F ATTCGAGCACAGATGACCAG; R AGGAGTC CGATATAGCTGTGG) *Apoc3* (F CTCACGACTCAATAGCTGGAG; R GGGCGTTGTCCAAACAGAAT) *Actb* (F‐GCAGCTCCTTCGTTGCCGGT; R‐TACAGCCCGGGGAGCATCGT). The efficiency of each primer set was determined from a four point standard curve and used to calculate relative expression using *Actb* as reference gene. Data are expressed as the fold‐change in relative expression relative to controls.

### 2.5 Hyperinsulinemic euglycemic clamps

Clamps were performed as previously described with minor modifications (Costa et al., 2016). Mice recovered for one week prior to clamp experiments following surgical implantation of an indwelling catheter in the right jugular vein. Mice were fasted 6h prior to infusion with 3‐^3^H‐glucose (Perkin Elmer) at a rate of 0.05 µCi min^−1^ for 120 min to determine basal glucose turnover. A primed infusion of insulin and 3‐^3^H‐glucose was administered at 7.14 mU kg^−1^ min^−1^ and 0.24 µCi/min, respectively, for 4 min, after which rates were reduced to 3 mU kg^−1^ min^−1^ insulin and 0.1 µCimin 3‐^3^H‐glucose. Blood was collected by tail massage for plasma measurements at set time points and a variable infusion of 20% dextrose was given to maintain euglycemia. Glucose turnover was calculated as the ratio of the 3‐^3^H‐glucose infusion rate to the specific activity of plasma glucose at the end of the basal infusion and during the last 40 min of the hyperinsulinemic infusion. Endogenous or hepatic glucose output represents the difference between the glucose infusion rate and the rate of glucose appearance.

### 2.6 Statistical analyses

Data are reported as the mean ± *SEM* and comparisons were made between WT and *Park2* KO groups within dietary treatments using two‐tailed, unpaired Student's *t*‐test. A *p*‐value of less than 0.05 was considered significant. Analyses were performed using GraphPad Prism 8.

## 3 RESULTS

Earlier work demonstrated improved glucose and pyruvate tolerance in *Park2* KO mice fed regular chow (RC); however, insulin sensitivity per se was not evaluated directly (Kim et al., 2011). We therefore performed hyperinsulinemic euglycemic clamps in WT and *Park2* KO mice fed RC for comparison with our previously reported HFD‐fed euglycemic clamp data (Costa et al., 2016; Figure 1a–c). Fasting plasma insulin levels were similar between genotypes on either diet and clamped plasma insulin levels were matched across groups in response to the hyperinsulinemic infusion (Costa et al., 2016; Figure 1a). There was no difference in the glucose infusion rate (GIR) required to maintain euglycemia comparing RC‐fed WT and *Park2* KO mice, demonstrating that whole‐body knockout of *Park2* had no effect on insulin sensitivity in RC‐fed mice (Figure 1b–c). In contrast, as previously reported, *Park2* KO mice were protected from HFD‐induced insulin resistance independent of changes in body weight after just one week of HFD feeding and the GIR required to maintain euglycemia was 1.8‐fold greater in *Park2* KO mice (Costa et al., 2016, Figure 1b–c). The improved insulin sensitivity was primarily due to changes in hepatic glucose production, where 60% of the difference in whole‐body insulin sensitivity was attributable to changes in hepatic glucose production and 40% to peripheral glucose uptake, but the mechanism for the observed changes in hepatic insulin action were not addressed (Costa et al., 2016). Insulin regulates hepatic glucose production directly by activating the insulin receptor signaling cascade, as well as indirectly by inhibiting adipose tissue lipolysis and the supply of fatty acids, and in turn acetyl‐CoA, to liver (Perry et al., 2015). There were no differences in basal or insulin‐stimulated plasma fatty acid levels comparing HFD‐fed WT and *Park2* KO mice during the clamp (Figure 1d), suggesting the changes in clamped hepatic glucose production were due to alterations in the direct, but not indirect effects of insulin on liver and thus, differences in hepatic insulin resistance.

Hepatic steatosis is associated with insulin resistance (Samuel & Shulman, 2016). We therefore measured liver triglyceride levels and found no difference between genotypes during RC feeding (Figure 2a), consistent with the lack of difference in insulin sensitivity during the euglycemic clamp (Figure 1). After one‐week HFD feeding, hepatic triglyceride levels were increased 1.4‐fold in WT mice compared with RC‐fed WT, but were unchanged in *Park2* KO mice and 40% less compared with HFD‐fed WT mice (Figure 2a; *p* < .05). Because of the lack of differences in whole‐body insulin sensitivity and liver steatosis between WT and *Park2* KO mice fed RC, we focused on HFD‐fed animals to understand the mechanistic basis for the differences in hepatic insulin sensitivity.

Accumulation of the intracellular lipid species diacylglycerol and ceramide are thought to contribute to hepatic insulin resistance by activating PKCε and protein phosphatase 2a (PP2a), respectively, resulting in inhibition of the insulin receptor signaling cascade (Petersen & Shulman, 2018). Diacylglycerol levels were 40% less in *Park2* KO compared with WT mice (Figure 2b, *p* < .01), which was primarily due to reduction in the membrane‐associated as opposed to cytosolic pool (Figure 2b, *p* < .01). In contrast, ceramide levels were modestly, but significantly increased in *Park2* KO compared with WT mice (Figure 2b; *p* < .05). Consistent with the reduction in membrane‐associated diacylglycerol levels, PKCε activity, expressed as the membrane‐to‐cytosolic ratio, was reduced by 40% in HFD‐fed *Park2* KO compared with WT mice (Figure 2c and d, *p* < .01).

In addition to subcellular localization, the acyl chain composition of diacylglycerol species may contribute to their ability to activate PKC isoforms and inhibit insulin signaling (Bergman, Hunerdosse, Kerege, Playdon, & Perreault, 2012). While several studies explored the relationship between diacylglycerol acyl chain composition and insulin resistance in skeletal muscle (Amati et al., 2011; Bergman et al., 2012; van Hees et al., 2011; Szendroedi et al., 2014), very few reports that addressed this question in liver exist. We therefore measured both acyl chain length and saturation in diacylglycerol species from liver cytosolic and membrane fractions, as well as from the total diacylglycerol pool (Figure 3). Within the cytosolic fraction, 12 of the 13 diacylglycerol species measured were reduced in HFD‐fed *Park2* KO compared with WT, but only four species (C18:1/C16:0, C18:2/C18:0, C18:1/C18:0, and C18:1/C18:1) were significantly less (Figure 3a; *p* < .05). Within the membrane fraction, all species of measured diacylglycerol were reduced in the *Park2* KO mice, and nine of 13 of the reduced species were significantly less compared with HFD‐fed WT (Figure 3b). Results from the total diacylglycerol pool were generally similar to the membrane fraction in that all species were reduced and nine of 13 species were significantly less (Figure 3c). When looking at which acyl chain species contributed to the overall reduction in diacylglycerol content within each fraction, the combined reduction in C18:1/C16:0, C18:1/C18:1, C18:1/C18:2, and C16:0/C18:2 accounted for ~90% of the difference within each fraction (Figure 3d). We also measured acyl chain composition of ceramide species in liver and found significant increases specifically in C22:0 (*p* < .05) and C24:0 (*p* < .001) ceramide and no differences in C16:0, C18:0, C20:0, or C24:1 species (Figure 3e).

Excessive endoplasmic reticulum (ER) stress and activation of the unfolded protein response (UPR) is also associated with hepatic insulin resistance (Hotamisligil, 2010). The UPR consists of three branches referred to by the names of the trans‐ER membrane proteins that sense an increased unfolded protein load within the ER and transduce this message to the cytosol, namely the inositol‐requiring enzyme 1 (IRE1α), PRK‐like eukaryotic initiation factor 2α kinase (PERK) and activating transcription factor 6 (ATF6) branches (Zhang & Kaufman, 2004). To determine if improved hepatic insulin sensitivity in HFD‐fed *Park2* KO mice was associated with changes in the ER stress response, we measured expression of a panel of transcripts downstream of each arm of the UPR. IRE1α possesses both kinase and ribonuclease activity, such that activation results in processing of the transcription factor X‐box‐binding protein‐1 (XBP1) and induction of a subset of ER stress response genes (Calfon et al., 2002; Lee, Iwakoshi, & Glimcher, 2003; Yoshida, Matsui, Yamamoto, Okada, & Mori, 2001). IRE1α/XBP1‐dependent genes *Dnajb9*, *Edem* and *Rpn1* were significantly reduced in HFD‐fed *Park2* KO liver, while *Sil1* was not (Figure 4a). Activation of PERK results in oligomerization and autophosphorylation, and in turn increased kinase activity (Ma, Vattem, & Wek, 2002). Activated PERK phosphorylates eukaryotic initiation factor 2α (eIF2α), resulting in translational arrest of the majority of transcripts; however, a small subset of mRNAs, including activating transcription factor 4 (*Atf4*) and its transcriptional targets are induced (Calfon et al., 2002). PERK‐mediated induction of *Atf4* expression was not different in HFD‐fed *Park2* KO compared with WT, while *Dnajc3* was significantly reduced and *Gadd34* was unchanged (Figure 4a). Activation of ATF6 results in its transport and cleavage in the Golgi, followed by migration of the cleaved form to the nucleus where it activates a subset of UPR genes (Haze, Yoshida, Yanagi, Yura, & Mori, 1999; Ye et al., 2000). ATF6‐dependent genes *Ero1β* and *Erp72* were significantly reduced in HFD‐fed *Park2* KO mice (Figure 4a).

In addition to lipotoxicity and ER stress, changes in local and systemic anti‐ and pro‐inflammatory cytokine levels are associated with insulin resistance (Glass & Olefsky, 2012). We therefore measured plasma levels of a panel of cytokines from WT and *Park2* KO mice, as well as a panel of obesity/diabetes‐related hormones produced by endocrine cells of the intestinal tract, pancreas and adipose tissue (Table 1). IL6 levels were two‐fold greater in RC‐fed *Park2* KO compared with WT mice (*p* < .05), and 11‐fold greater in HFD‐fed *Park2* KO compared with WT mice (*p* < .05; Table 1). IL10 and TNFα levels were significantly greater in HFD‐fed *Park2* KO compared with WT mice (two‐fold, *p* < .01 and 1.7‐fold, *p* < .001, respectively; Table 1), while there were no differences in interferon γ (IFNγ), interleukin 2 (IL2), interleukin 5 (IL5) or C‐X‐C motif chemokine ligand 1 (CXCL1) between genotypes on either diet (Table 1). Of the seven obesity/diabetes‐related hormones measured, leptin was the only factor that differed between *Park2* KO and WT mice on both diets, where plasma levels were 65% and 94% less in RC‐ and HFD‐fed *Park2* KO compared with WT mice, respectively (*p* < .05, *p* < .001; Table 1). There was a modest, but insignificant reduction in plasma resistin levels comparing HFD‐fed *Park2* KO to WT mice (*p* = .08), and no detectable differences in glucagon‐like peptide 1 (GLP1), glucagon, gastric inhibitory peptide (GIP), plasminogen activator inhibitor 1 (PAI1), or ghrelin levels (Table 1).

Next, we measured phosphorylation and activation of kinases downstream of the UPR and inflammatory signaling pathways that are implicated in the pathogenesis of hepatic insulin resistance (Hotamisligil, 2010). IRE1α‐mediated activation of c‐Jun NH_2_‐terminal kinase (JNK) is thought to contribute to hepatic insulin resistance via phosphorylation and inhibition of insulin receptor substrate 1 (IRS1) (Ozcan et al., 2004). Despite an apparent decrease in the IRE1α arm of the UPR in HFD‐fed *Park2* KO mice (Figure 4a), there was no difference in JNK phosphorylation, indicative of activation, between groups (Figure 4b). Phosphorylation and activation of the Iκβ kinase (IKKβ) via UPR and inflammatory cytokine signaling is also proposed to induce hepatic insulin resistance (Arkan et al., 2005; Cai et al., 2005); however, we found no differences in IKKβ phosphorylation between groups (Figure 4c).

As mentioned earlier, *Park2* KO mice fed HFD for six and a half weeks were protected from obesity and subsequently hepatic steatosis (Kim et al., 2011). We previously demonstrated that hepatic fatty acid transport, esterification and oxidation were unchanged in *Park2* KO compared with WT mice (Costa et al., 2016), suggesting that changes in energy balance that manifest at the level of the hepatocyte during short‐term HFD feeding, prior to changes in body weight, contributed to the reduced steatosis observed here. To test this idea, we measured adenylate levels, as well as activation of the cellular energy sensor AMPK in liver of HFD‐fed *Park2* KO and WT mice (Figure 5). Total adenylate levels were 12% less in *Park2* KO mice (*p* < .01), which was the result of a 30% reduction in ATP and a modest, but non‐significant increase (15%) in AMP and no change in ADP (Figure 5a). The combined increase in AMP and decrease in ATP resulted in an approximate twofold increase in the AMP to ATP ratio in HFD‐fed *Park2* KO liver compared with WT mice (*p* < .05; Figure 5b). AMPK is subject to multiple modes of regulation, most notably allosteric activation by AMP during times of increased ATP consumption that increases the cellular AMP/ATP ratio, facilitating phosphorylation of a conserved threonine residue (T172) in the AMPKα subunit and stimulation of AMPK's kinase activity (Gowans, Hawley, Ross, & Hardie, 2013). Consistent with the observed increase in the AMP/ATP ratio, AMPKα T172 phosphorylation was 2.2‐fold greater in *Park2* KO compared with WT mice (*p* < .05; Figure 5c–d). AMPK is a potent inhibitor of acetyl‐CoA carboxylase (ACC), the enzyme that converts acetyl‐CoA to malonyl‐CoA. Malonyl‐CoA levels were 30% less in *Park2* KO mice (*p* < .05; Figure 5e). Lastly, we measured expression of a panel of transcripts that are typically suppressed in liver in response to AMPK activation (Viollet et al., 2006) (Figure 5f). There was no difference in *Pepck* expression, while *Fasn*, *Hnf4α*, *Srepb1*, *Apob*, and *Apoc3* mRNA levels were all significantly reduced (*p* < .05 ‐ *p* < .001; Figure 5f).

## 4 DISCUSSION

Here we provide the first direct evidence demonstrating no change in whole‐body insulin sensitivity in RC‐fed *Park2* KO mice, as well as mechanistic insight with regard to the improved hepatic insulin sensitivity in short‐term HFD‐fed *Park2* KO mice. We find that after one‐week HFD feeding, *Park2* KO mice are protected against hepatic steatosis and diacylglycerol accumulation, and that hepatic PKCε activation is subsequently reduced. Liver ceramide levels, particularly C24:0 species, which are negatively associated with hepatic insulin resistance (Montgomery et al., 2016; Park et al., 2013; Raichur et al., 2014), were increased in HFD‐fed *Park2* KO mice. TNFα and IL6, which are both positively associated with insulin resistance (Hotamisligil, Shargill, & Spiegelman, 1993; Pickup, Mattock, Chusney, & Burt, 1997), were increased in plasma of HFD‐fed *Park2* KO mice, inconsistent with the observed phenotype with regards to insulin sensitivity. We also observed increased plasma levels of IL10, which has been shown to inhibit the effects of IL6 with regards to insulin resistance (Kim et al., 2004), and may have contributed to the improved insulin sensitivity. Although body weight was not different in HFD‐fed WT compared with *Park2* KO mice, fat mass and plasma leptin levels were reduced in *Park2* KO mice, and may have contributed to the improved hepatic insulin sensitivity (Costa et al., 2016). Finally, transcriptional markers of the ER stress response were also reduced in HFD‐fed *Park2* KO mice, but there was no detectable difference in downstream activation of JNK or IKKβ between groups. Together, our data suggest that reduced lipotoxicity in liver of HFD‐fed *Park2* KO mice contributed to their improved hepatic insulin sensitivity, and that changes in very‐long‐chain ceramides, ER stress‐ or IL10‐mediated signaling pathways may have contributed, as well.

A large number of studies in both rodents and humans reported a positive relationship between intracellular diacylglycerol content and insulin resistance in both skeletal muscle and liver (Samuel & Shulman, 2016), similar to what we observed here. In addition to total diacylglycerol content, the localization of diacylglycerol species may influence their ability to activate novel PKC (nPKC) isoforms and inhibit insulin signaling (Petersen & Shulman, 2018). For example, hepatic knockdown of comparative gene identification‐58 (CGI‐58), a lipid droplet‐associated protein that serves as an activating co‐factor for adipose triglyceride lipase, results in diacylglyerol accumulation, yet preserved hepatic insulin sensitivity (Brown et al., 2010; Cantley et al., 2013). In contrast to typical HFD‐fed mice, where diacylglycerol accumulates in the membrane fraction, diacylglycerol in CGI‐58 knockdown mice accumulated in the lipid droplet or lipid‐associated ER fraction of the cell (Cantley et al., 2013). PCKε was similarly localized to the lipid droplet/ER compartment of CGI‐58 knockdown mice, suggesting that redistribution of PKCε away from compartments of the cell involved in insulin receptor signaling, that is the membrane associated fraction, may account for the dissociation between hepatic diacylglycerol content and insulin resistance (Cantley et al., 2013). Thus, our observation of reduced membrane‐associated diacylglycerol and PKCε in HFD‐fed *Park2* KO mice is consistent with their improved hepatic insulin sensitivity (Costa et al., 2016). In addition to localization, the acyl chain composition of diacylglycerol species may contribute to their ability to activate nPKC isoforms (Madani, Hichami, Legrand, Belleville, & Khan, 2001). For example, studies in human subjects reported a positive association between saturated fatty acid diacylglycerol species and insulin resistance (Bergman et al., 2012, 2010; van Hees et al., 2011), whereas others have reported a negative association (Amati et al., 2011) or no relationship at all (Coen et al., 2010). Far fewer studies have explored the relationship between acyl chain composition of diacylglycerols and insulin resistance in liver. Within the one reported study of which we are aware, a generalized increase in most diacylglycerol species measured was observed in association with hepatic insulin resistance (Cantley et al., 2013). This is consistent with our data here, where all sub‐species of diacylglycerol measured in the membrane fraction were reduced in HFD‐fed *Park2* KO mice, suggesting acyl chain composition of diacylglycerol species does not specifically contribute to differences in insulin sensitivity in HFD‐fed rodent models. Interestingly, reductions in just four acyl chain species, specifically C18:1/C16, C18:1/C18:1, C18:1/C18:2, and C16/C18:2 accounted for 90% of the reduction in total diacylglycerol within each measured cellular fraction. These same species of fatty acids account for approximately 93% of the fatty acid species found in the HFD used, suggesting hepatic diacylglycerol content is largely reflective of dietary composition.

In addition to diacylglyerol, increases in hepatic ceramide levels, particularly long‐chain ceramides composed of C16‐C22 fatty acids, are positively associated with insulin resistance (Meikle & Summers, 2017; Montgomery et al., 2016; Raichur et al., 2014). Interestingly, reductions in hepatic very‐long‐chain ceramides, such as C22:0 and C24:0, are negatively associated with insulin resistance (Montgomery et al., 2016; Park et al., 2013; Raichur et al., 2014) and may confer protection from lipid‐induced insulin resistance by modulating the ER stress response or by increasing β‐oxidation (Montgomery et al., 2016; Raichur et al., 2014). Using a liver lipidomics approach, Montgomery and colleagues measured ceramide species in five commonly studied mouse strains and found that reductions in very‐long‐chain species of C22 or greater, as well as expression of the enzyme that catalyzes their synthesis, ceramide synthase 2 (*Cers2*), occurred in strains susceptible to diet‐induced insulin resistance (Montgomery et al., 2016). Overexpression of *Cers2* in mouse primary hepatocytes increased C22:0 and C24:0 ceramides species and conferred protection from lipid‐induced insulin resistance, which was associated with reduced markers of ER stress and no differences in JNK activation, consistent with our observations here. Similarly, Raichur and colleagues observed that reductions in very‐long‐chain ceramide levels in *Cers2* haploinsufficient mice were associated with increased susceptibility to lipid‐induced insulin resistance and reduced hepatic fatty acid oxidation (Raichur et al., 2014). Increases in hepatic C22:0 and C24:0 in HFD‐fed *Park2* KO mice may therefore contribute to their protection from insulin resistance by modulating the ER stress response or by promoting lipid oxidation to prevent accumulation of long‐chain ceramide species or membrane‐associated diacylglycerols.

Increased plasma levels of TNFα and IL6 are associated with insulin resistance (Shoelson, Herrero, & Naaz, 2007), inconsistent to observations made here where HFD‐fed *Park2* KO mice were more insulin sensitive despite increased plasma TNFα and IL6 levels. One possibility is that the differences observed here, although significantly different statistically, may not be significant biologically given the low absolute levels of cytokines measured, although the levels we detected are similar to previous reports (Dandona et al., 1998; Kern, Ranganathan, Li, Wood, & Ranganathan, 2001; Vozarova et al., 2001). Another possibility is that the parallel increase in the anti‐inflammatory cytokine, IL10, which was increased twofold in HFD *Park2* KO mice, offset the effects of the pro‐inflammatory cytokines, which was shown to occur previously with regard to IL6 (Kim et al., 2004). While the observed changes in plasma cytokines in our model may not relate to the hepatic insulin resistance phenotype, they provide additional support for recent insight into how loss of Parkin‐mediated mitophagy contributes to disease pathogenesis. Recently, a new model for how *Park2* mutations and loss of mitophagy give rise to Parkinson's disease was proposed where in the absence of mitochondrial quality control, mitochondrial DNA is released from damaged mitochondria and acts as a pro‐inflammatory signal, driving local and systemic inflammation and subsequent neurodegeneration (Sliter et al., 2018). Within this model, both acute and chronic mitochondrial stressors induced a marked increase in plasma IL6 levels on the order of 1000‐fold above baseline in *Park2* KO compared with WT mice (Sliter et al., 2018). Here we observed a similar, but more moderate induction of IL6 on the order of 10‐fold above baseline in HFD‐fed *Park2* KO mice, as well as a twofold increase in RC‐fed animals. Our data suggest that even in the absence of a specific mitochondrial stressor, deletion of *Park2* is associated with activation of an immune response and increased IL6 levels, and furthermore, that short‐term HFD feeding exacerbates this effect. Given the specific effects of a short‐term HFD on hepatic lipid accumulation and insulin resistance (Samuel et al., 2004; Turner et al., 2013), we predict that the source of the pro‐inflammatory signal in the absence of *Park2* to be the liver, but this supposition requires further testing.

Given previous work demonstrating that pharmacological or genetic activation of hepatic AMPK in nutritionally stressed rodents can reduce liver triglyceride levels and improve glucose homeostasis (Cool et al., 2006; Woods et al., 2017), it is tempting to speculate that the increased AMPK activation in the HFD‐fed *Park2* KO mice contributed to their improved hepatic phenotype. While we cannot rule out this possibility, the lack of differences in fatty acid uptake, oxidation or triglyceride accumulation we observed when comparing WT and *Park2* KO primary hepatocytes argues against this interpretation (Costa et al., 2016). Instead, we propose that hepatic activation of AMPK reflects negative energy balance in the *Park2* KO during HFD feeding that manifests over the long‐term as reduced body weight and adiposity compared with WT mice (Kim et al., 2011). Here over the short‐term, this same mechanism presents as reduced liver fat and improved hepatic insulin sensitivity. The underlying mechanism for the negative energy balance and protection from diet‐induced obesity in the *Park2* KO mice remains undetermined. One unexplored explanation for this phenomenon within the current study is that changes in mitochondrial function, specifically oxidative phosphorylation, in response to loss of *Park2*‐mediated mitophagy contributed to the reduced hepatic energy charge. Previously, liver‐specific deletion of the electron transport chain assembly factor, apoptosis inducing factor (*Aif*), resulted in loss of mitochondrial complex I and ATP synthase activities. Impaired oxidative phosphorylation within this model was associated with reduced hepatic ATP levels, activation of AMPK and protection from diet‐induced obesity, similar to *Park2* KO mice (Pospisilik et al., 2007). Another possibility is that increased dark cycle activity, which was reported in *Park2* KO mice fed HFD for one or six and a half weeks, contributed to their energy deficit (Costa et al., 2016; Kim et al., 2011). However, no differences in energy expenditure were detected in the short‐term HFD challenge study and although energy expenditure was reportedly increased in the long‐term study, correction of the data to body weight when the models being compared differed significantly in terms of adiposity makes interpretation of the result difficult (Kaiyala & Schwartz, 2011; Kim et al., 2011). And although we demonstrated reduced intestinal lipid absorption in the *Park2* KO mice, the amount of lipid detected in feces was quantitatively insufficient to account for the protection from diet‐induced obesity (Costa et al., 2016). Another possibility is that the kinetics of intestinal lipid absorption may differ in *Park2* KO mice and contribute to the phenotype, as has been reported in Diacylglycerol o‐acyltransferase‐1 (*Dgat1*) and Acyl CoA:monoacylglycerol acyltransferase‐2 (*Mgat2*) KO mice (Smith et al., 2000; Yen et al., 2009). This is an intriguing hypothesis given *Park2's* role in Parkinson's disease where *Park2* mutations give rise to loss of dopaminergic neurons in the central nervous system, begging the question as to whether similar changes in dopamine or other monoamine producing neurons in the enteric nervous system occur in *Park2* KO mice during HFD feeding and thus alter intestinal motility.

In summary, we provide evidence that reduced hepatic energy balance in short‐term HFD‐fed *Park2* KO mice is associated with reduced hepatosteatosis and improved hepatic insulin sensitivity. We provide further evidence supporting the hypotheses that differences in membrane‐associated diacylglycerol levels and PKCε activity, and very‐long‐chain ceramide species, contribute to alterations in hepatic insulin sensitivity. Finally, we report confirmatory evidence supporting the recent observation that loss of *Park2* is associated with a pro‐inflammatory state, particularly increases in plasma IL6 levels under conditions of mitochondrial stress, suggesting that even short‐term HFD feeding presents a significant stress to hepatic mitochondria. Future studies exploring changes in hepatic mitochondrial respiratory chain function, as well as the role of *Park2* in the enteric nervous system and in gastric motility may provide insight into the still unresolved mechanism for reduced energy homeostasis in *Park2* KO mice during HFD‐feeding.

## CONFLICT OF INTEREST

The authors have no conflicts of interest to disclose.
